# Nationwide Implementation of a Digital Health Module for Chronic Kidney Disease Screening: A RE-AIM Evaluation in Peru’s Social Health System

**DOI:** 10.3390/medsci14030373

**Published:** 2026-07-04

**Authors:** Percy Allan Vidal Orbegozo, Lizbeth Carmen Arce Gallo, Isabel Julia Alamo Palomino, Madelaine Huanca Roca, Juana Eliza Ormeño Galarza, Dayana Ticona-Tiña, Luis Randy Loayza Arroyo, Alexis German Murillo Carrasco, Moisés Apolaya-Segura, Daysi Zulema Diaz-Obregón

**Affiliations:** 1 Centro Nacional de Salud Renal, Seguro Social de Salud (EsSalud), Lima 15073, Peru; allanvip79@gmail.com (P.A.V.O.); lizarceg@gmail.com (L.C.A.G.); isabel.alamo7@gmail.com (I.J.A.P.); madehuanca@hotmail.com (M.H.R.); juana_eog@hotmail.com (J.E.O.G.); 2 ONG Innovation and Science for the Care and Support of Society–INNOVACARE, Lima 15023, Peru; dayana.ticona.29@gmail.com (D.T.-T.); lrandy.loayza02@gmail.com (L.R.L.A.); daysi.diaz@essalud.gob.pe (D.Z.D.-O.); 3 Organization for Medical Innovation and Collaboration for Sciences—OMICS, Lima 15072, Peru; 4 Instituto de Evaluación de Tecnologías en Salud e Investigación (IETSI), Seguro Social de Salud (EsSalud), Lima 15073, Peru; moises.apolaya@gmail.com

**Keywords:** chronic kidney disease, screening, digital tools, MOSARE, RE-AIM, EsSalud

## Abstract

**Background:** Chronic kidney disease (CKD) is a major public health problem, primarily affecting patients with diabetes mellitus and/or hypertension. Early detection is critical to delay progression to renal replacement therapy. Therefore, this study aims to evaluate the implementation of a digital module for CKD detection in at-risk patients within the Peruvian Social Health Insurance system (EsSalud) during 2024. **Materials and Methods:** Data were collected through the Renal Health Module (MOSARE), a digital tool integrated into EsSalud’s electronic health record for monitoring patients at risk of CKD. Key indicators were assessed using the RE-AIM framework: reach, effectiveness, adoption, implementation, and maintenance. Quantitative analyses evaluated patient identification, screening, and diagnosis, while qualitative analyses of technical documents identified barriers, facilitators, and stakeholder perceptions. **Results:** In 2024, MOSARE was implemented in a population of 1,667,856 insured individuals with CKD-related risk conditions, predominantly women (55.7%) and adults aged >55 years (88.3%). Nationwide, 93,266 patients were screened (5.59% reach), with 34.3% diagnosed with CKD. Screening coverage showed substantial geographic variability (0.35–29.59%). A total of 32,004 CKD cases were identified, with 84.2% in early stages (1–3A). Adoption reached 66.8% of level I and II healthcare facilities, with variability across networks. Regarding maintenance, 65% of trained facilities sustained active use. Implementation gaps were associated with interoperability issues, resource limitations, and training variability. **Conclusions:** MOSARE proved to be a feasible and operationally sensitive tool for CKD screening and risk identification, improving integration of renal care. However, addressing technical, training, and resource barriers is essential to ensure sustainability and scalability.

## 1. Introduction

Chronic kidney disease (CKD) affects approximately 11% to 13% of the global population, with more than 800 million people diagnosed, and represents one of the leading causes of morbidity and mortality worldwide [1]. This condition, primarily associated with risk factors such as diabetes mellitus and hypertension, accounts for over 70% of CKD cases and accelerates progression to advanced stages [2,3]. Moreover, more than 40% of cases worldwide are not diagnosed at early stages, leading to increased hospitalization rates and a significant decline in patients’ quality of life [4].

In Latin America, CKD remains a growing public health concern, contributing to increased mortality and loss of healthy life years [5]. This has prompted the implementation of secondary prevention strategies and comprehensive CKD management approaches, which have proven to be cost-effective by reducing progression to dialysis and mortality, yielding an average saving of USD 783 per patient and gains of 0.36 dialysis-free years and 0.04 quality-adjusted life years (QALYs) [6].

In recent years, various digital tools have been developed to improve screening and early diagnosis of chronic diseases such as CKD. These include integrated systems that enable remote monitoring and systematic use of laboratory biomarkers, such as estimated glomerular filtration rate (eGFR), urine albumin-to-creatinine ratio (ACR), and serum creatinine and potassium levels—facilitating more timely and accurate diagnoses in outpatient settings [7]. Additionally, emerging technologies have enabled non-invasive testing through the analysis of body fluids, increasing accessibility and reducing reliance on centralized laboratories [8,9].

Among these innovations, the Renal Health Module (MOSARE) was developed by EsSalud as an institutional, population-level digital health tool integrated into the nationwide electronic health record system (EsSI). MOSARE supports the screening of the general insured population based on age criteria, as well as the targeted identification of individuals at high risk for CKD. It automates the calculation of key renal indicators such as estimated glomerular filtration rate (eGFR) and albumin-to-creatinine ratio (ACR), facilitates CKD staging according to KDIGO criteria, and generates automated alerts for clinical follow-up. By combining universal digital triggers with routine health information systems, MOSARE represents one of the first large-scale digital nephrology initiatives designed to capture both the broader eligible population and specific clinical risk groups within the Peruvian social health insurance system.

These innovations highlight the potential of digital solutions to transform early detection in public health; however, their impact and feasibility require rigorous evaluation using conceptual frameworks such as RE-AIM to ensure effectiveness, sustainability, and scalability across different contexts [10,11,12,13].

Despite the growing adoption of digital health solutions, evidence regarding their implementation, adoption, sustainability, and real-world performance for CKD screening in middle-income countries remains limited. Furthermore, large healthcare systems frequently face challenges related to interoperability, workforce training, heterogeneous infrastructure, and integration into routine clinical workflows. Evaluating these dimensions is essential to determine whether digital innovations can effectively contribute to strengthening CKD prevention and management at scale.

The Peruvian Social Health Insurance system (EsSalud) is a key institution that serves more than 30% of the national population and operates the Intelligent Health Services System (EsSI), a nationwide electronic health record. Despite this, challenges remain in integrating digital tools to optimize timely diagnosis and preventive monitoring, underscoring the need for innovative and scalable CKD solutions [14,15]. Therefore, this study aims to evaluate the implementation of the Renal Health Module (MOSARE) for the identification, detection, and monitoring of CKD in at-risk patients within EsSalud, using the RE-AIM framework as the analytical approach.

## 2. Materials and Methods

### 2.1. Study Design

We conducted a convergent parallel mixed-methods implementation study to evaluate the real-world performance of the Renal Health Module (MOSARE) within EsSalud at the end of its first fiscal year (2024). The study design is explicitly operationalized through the RE-AIM conceptual framework (Reach, Effectiveness, Adoption, Implementation, Maintenance) [10,11], nesting both quantitative and qualitative methods to ensure methodological transparency:

- Quantitative Component: An observational, retrospective, registry-based cohort design was used to analyze secondary clinical and administrative data. This component quantified indicators for Reach (eligible vs. screened patients), Effectiveness (CKD staging and risk stratification via KFRE), and Implementation (fidelity to screening protocols across networks).
- Qualitative Component: A descriptive qualitative design using reflexive thematic analysis was employed to evaluate institutional technical reports, operational directives, and implementation meeting minutes. This component captured contextual dimensions of Adoption (barriers and facilitators for active system integration at the facility level) and Maintenance (sustainability factors and long-term policy integration).

No formal a priori sample size calculation or statistical power estimation was performed, as this study adopted a census-based approach utilizing the entire nationwide electronic health record registry available within the EsSI platform for the 2024 year. The final study size (*N* = 4,613,598 for the macro-eligible universe; *n* = 93,368 for the actively screened cohort) was determined strictly by data availability and inclusion completeness. By analyzing the comprehensive institutional universe rather than a probabilistic sample, we minimized sampling error and maximized the statistical precision and generalizability of the findings across all regional healthcare networks within EsSalud.

### 2.2. Context and Intervention

System-Level Context: The Peruvian Social Health Insurance system (EsSalud) provides care to over 11 million insured individuals through an integrated network using a centralized electronic health record system (EsSI). Persistent gaps in chronic disease detection drove the institutional deployment of MOSARE.

Digital System Capabilities and Automation: MOSARE is a digital health module embedded directly within the EsSI platform. Its level of automation includes: (1) automated screening triggers that flag eligible patients based on electronic health records, (2) automated calculation of eGFR (using the CKD-EPI equation) and ACR upon laboratory data entry, and (3) an automated clinical decision-support function that populates a color-coded alert for CKD staging according to KDIGO criteria.

Healthcare Process Intervention: Guided by Directive No. 018-GCPS-ESSALUD-2020, the clinical workflow is operationalized in active consultation. When a physician or nurse visualizes MOSARE’s automated alert, they are required to order targeted laboratory follow-up, initiate standardized early-stage nephroprotection protocols, or execute referral pathways for advanced stages.

### 2.3. Study Population and Variables

The target population encompassed the entire universe of insured individuals nationwide registered within the EsSI electronic health record system who triggered MOSARE’s automated baseline eligibility evaluation during the 2024 fiscal year (*N* = 4,613,598). This institutional denominator includes individuals systematically categorized by the system for screening eligibility based on age cohorts, administrative enrollment records, or pre-existing cardiometabolic risk conditions. Within this macro-eligible universe, the active screened study population consisted of outpatients attending level I and II healthcare facilities nationwide from January to December 2024 who had complete laboratory data (serum creatinine, microalbuminuria, and/or creatinuria) successfully processed and recorded in the MOSARE platform (*n* = 93,368), after excluding duplicated and technically inconsistent records.

It is critical to note that due to the retrospective, registry-based nature of this first-year evaluation, CKD staging was determined based on the automated calculation of the index laboratory encounter processed within MOSARE during 2024. Consequently, the reported cases represent presumptive positive screenings or newly identified risk stages, as longitudinal confirmation of chronicity (the conventional 3-month persistence criteria stipulated by KDIGO) could not be systematically verified across all healthcare networks within this initial fiscal cycle.

Sociodemographic, clinical, laboratory, and implementation variables were obtained from the MOSARE system. Sociodemographic variables included age, sex, geographic location (department and healthcare network), and facility level. Clinical and laboratory variables included serum creatinine, urine albumin, albumin-to-creatinine ratio, and estimated glomerular filtration rate (eGFR) calculated using the CKD-EPI equation, which was used to classify CKD stages according to KDIGO criteria. Additionally, the risk of progression to kidney failure at 2 and 5 years was estimated using the Kidney Failure Risk Equation (KFRE) [16].

Implementation variables were assessed using the RE-AIM framework [12,13], allowing the identification of barriers and facilitators for integrating the intervention into the health system. The evaluated dimensions were:

- Reach: Defined as the proportion of adults with CKD risk factors (diabetes, hypertension, or age > 55 years) registered in MOSARE relative to the total at-risk population within EsSalud [7]. Analyses were conducted by geographic macro-regions (Lima-East, South, North, and Center) and stratified by age and sex.
- Effectiveness: Assessed the impact of MOSARE on screening and timely CKD stage identification. Indicators included newly identified cases and distribution by disease stage, compared with historical data. Additionally, MOSARE enabled estimation of CKD progression risk using KFRE [16].
- Adoption: Defined as the proportion of healthcare facilities (IPRESS) implementing MOSARE. To evaluate the Adoption and Maintenance dimensions of the RE-AIM framework, a structured documentary review was conducted using institutional implementation reports, technical monitoring documents, and implementation meeting minutes generated during the nationwide deployment of MOSARE. Documents were systematically reviewed to identify evidence related to acceptance of the intervention, barriers and facilitators to implementation, integration into healthcare processes, and sustainability over time. Extracted information was organized into predefined thematic categories corresponding to the Adoption and Maintenance domains of the RE-AIM framework, providing complementary contextual information to support interpretation of the quantitative findings.
- Implementation: Evaluated fidelity to screening, diagnosis, and initial management protocols, including early-stage detection rates and identification of high-risk patients using KFRE. Time from implementation to first CKD diagnosis was also analyzed.
- Maintenance: Assessed sustainability based on continued system use, technical support activities, ongoing training, and operational monitoring, as well as organizational and technical factors influencing long-term integration.

### 2.4. Statistical Analysis

Data were collected through direct extraction from MOSARE and complemented with technical reports from the National Renal Health Center at EsSalud. Descriptive statistics were used to estimate proportions, and non-parametric tests were applied to compare regions. Qualitative analysis used coding approaches to identify patterns related to barriers and facilitators. Analyses were performed using Stata 12.0 with a 95% significance level.

### 2.5. Methodological Biases

To ensure the validity of our RE-AIM evaluation, we systematically addressed potential sources of bias inherent to registry-based secondary data. First, ascertainment bias (selection bias) represents a potential limitation, as data extraction relied on individuals actively utilizing healthcare services within the EsSI system. The direction of this bias likely overestimates the true population-level prevalence of CKD within the screened cohort (*n* = 93,368), given that symptomatic or higher-risk patients are more prone to visit clinics. However, its magnitude is considered moderate, as the massive eligible universe (*N* = 4,613,598) balances out specific outpatient skewness.

**Figure 1:**
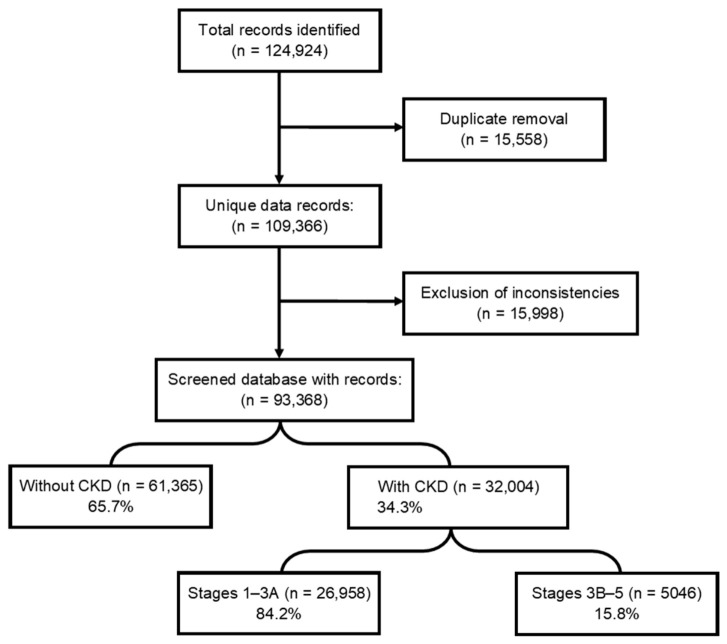
Flowchart of patients with CKD risk factors registered in MOSARE at level I and II healthcare facilities of the Social Health Insurance system, 2024. The duplicate filter retained the first and most complete record; the inconsistency filter excluded records with screening results not consistent with staging according to their code; and the laboratory filter removed records with non-coherent laboratory values (e.g., null or negative values).

Second, information bias could arise from incomplete electronic records or laboratory entry omissions. As shown in our selection flowchart (Figure 1), 15,998 inconsistent records were systematically excluded. The direction of this bias potentially underestimates the operational ‘Reach’ and ‘Adoption’ indicators of the module. Its magnitude is classified as low, representing less than 13% of the total identified records (*n* = 124,924), thereby preserving the structural integrity and interpretability of the national findings.

## 3. Results

### 3.1. Population Characteristics

During the study period, 124,924 individuals were registered for screening through the MOSARE platform. Of these, 15,558 records were excluded due to duplication and incomplete data, while an additional 15,998 records were discarded for not meeting laboratory criteria or presenting data inconsistencies. Consequently, the final analysis included 93,369 unique patient records. Of the total screened population, 34.3% (*N* = 32,004) were classified into one of the CKD stages (Figure 1).

**Figure 2:**
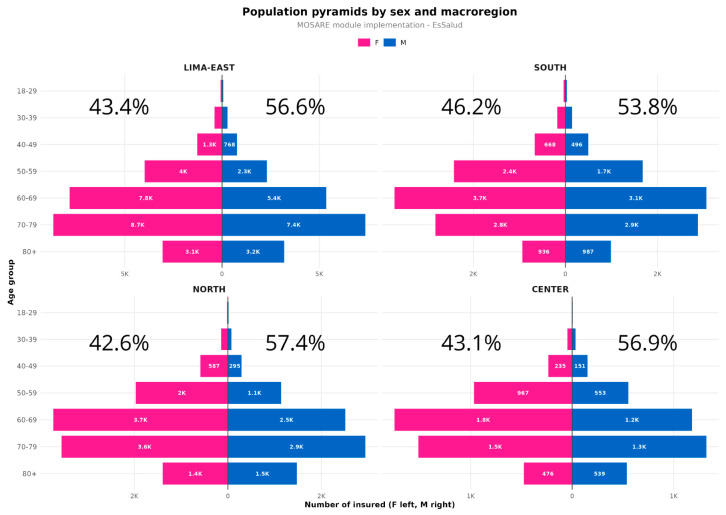
Population pyramid of patients registered in MOSARE by macro-regions within the Social Health Insurance system, 2024.

Table 1 and Figure 2 describe the demographic and clinical characteristics of the population at risk for CKD included in MOSARE during 2024 (*N* = 1,667,856). Women represented 55.3% of the at-risk population, while men accounted for 44.7%. The population was predominantly composed of older adults (>55 years), who represented 88.3% of all individuals at risk, whereas adults aged 30–55 years accounted for 11.4%. Regarding comorbidities, hypertension was present in 35.9% of the population, diabetes in 20.6%, and the coexistence of both conditions in 10.2%. Overall, these findings indicate that the population targeted by MOSARE is highly concentrated among older adults with cardiometabolic risk factors, reinforcing the need for systematic CKD screening strategies in primary care settings.

### 3.2. Reach

Table 2 presents the distribution of patients screened through MOSARE according to department and CKD status during 2024. Nationwide, 93,273 patients with CKD risk factors were screened, representing 5.59% of the total population at risk registered in the Social Health Insurance system (*N* = 1,667,856). Of all screened individuals, 34.3% (*n* = 31,990) were diagnosed with CKD, while 65.7% (*n* = 61,283) did not meet diagnostic criteria. Screening coverage varied considerably across departments, ranging from 0.35% in Tumbes to 29.59% in Tacna. Higher coverage levels were observed in Tacna (29.59%), Callao (25.72%), Ucayali (13.36%), Huánuco (11.11%), Loreto (11.03%), Lambayeque (10.32%), and Puno (10.32%), whereas lower coverage was identified in Tumbes (0.35%), Ica (0.74%), Huancavelica (1.42%), La Libertad (1.70%), and Cusco (1.82%).

Although Lima accounted for the largest absolute number of screened individuals (30,327 patients), its proportional reach was only 4.35%, suggesting substantial opportunities to expand screening activities in highly populated regions. Similarly, Callao achieved both a high screening volume and high coverage, indicating successful implementation of the MOSARE strategy in that network. Overall, the findings reveal marked geographic heterogeneity in screening performance across the country (Table 2).

**Figure 3:**
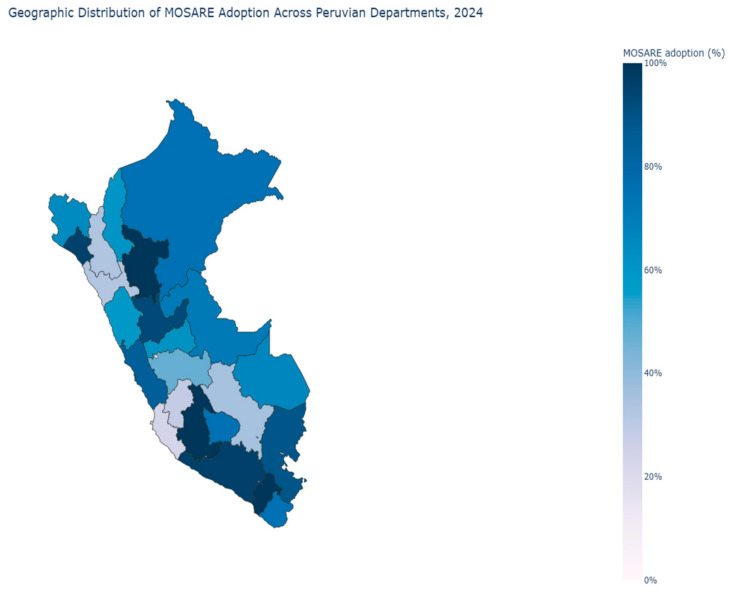
Proportion of level I and II healthcare facilities using MOSARE, according to healthcare network within the Social Health Insurance system, 2024.

Marked geographic variability was observed, ranging from 0.11% in Tumbes to 10.41% in Tacna. Higher coverage was found in Loreto (3.98%), Ucayali (3.58%), Moquegua (3.51%), and Lambayeque (3.40%), while lower coverage was observed in Ica (0.24%), Huancavelica (0.31%), Cajamarca (0.64%), and La Libertad (0.64%). Although Lima and Callao accounted for the largest number of screened patients, their relative reach was moderate (2.5% and 1.82%, respectively), suggesting limitations in coverage given the size of the at-risk population. Overall, screening distribution was heterogeneous both in volume and coverage. Additionally, 68.46% of screened patients were identified in level I and II facilities, highlighting the role of primary care in early detection (Figure 3).

### 3.3. Effectiveness

**Figure 4:**
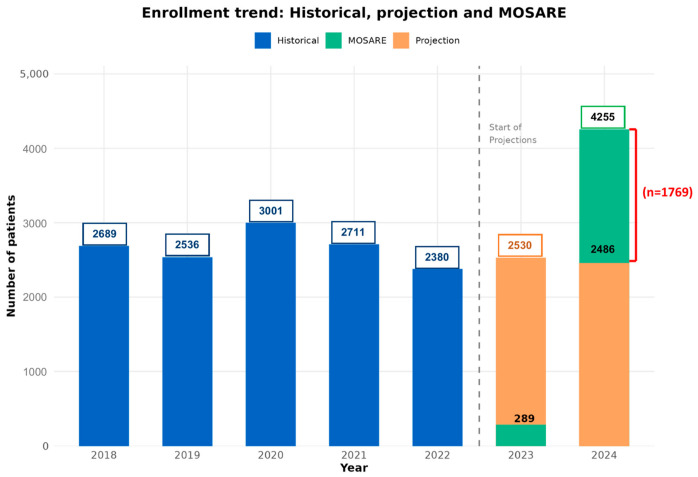
Detection of CKD cases in stages 3B to 5 following MOSARE implementation compared with the historical projection based on RENDES (2018–2024).

During the evaluation period, 32,004 CKD cases were diagnosed, of which 26,958 (84.2%) were in stages 1 to 3A and 5046 (15.8%) in stages 3B to 5 (Figure 1). Figure 4 illustrates trends in advanced CKD (stages 3B–5) from 2018 to 2024. The number of advanced CKD cases identified through MOSARE exceeded the historical projections derived from EsSalud National Dialysis Registry (RENDES) records by 1769 cases. However, this difference should be interpreted cautiously, as variations in screening intensity, reporting practices, case ascertainment, and temporal factors may have contributed to the observed increase.

Screening also enabled estimation of progression risk using the Kidney Failure Risk Equation (KFRE), showing cumulative risks of 6.48% at 2 years and 10.41% at 5 years. Figure 4 presents the time from indication to initiation of renal replacement therapy, with a mean of 58 days, reflecting timely management within the MOSARE system.

### 3.4. Adoption

MOSARE was implemented in 245 out of 367 level I and II healthcare facilities, achieving 66.8% coverage in 2024. Adoption varied widely, from 23.5% in Ica to 100% in Moquegua, Ayacucho, and Moyobamba. High adoption (>80%) was observed in Lima networks (Rebagliati, Sabogal, Almenara), whereas lower adoption occurred in Tumbes, Cajamarca, La Libertad, Cusco, and Ica. Barriers included limited knowledge of CKD guidelines and interoperability issues with electronic health records, particularly in lower-resource settings. Successful experiences in Lima networks demonstrated the importance of technical support and clinical leadership (Table 3).

### 3.5. Implementation

MOSARE enabled the identification and staging of 32,004 CKD cases across the Social Health Insurance system. Overall, early and intermediate stages predominated, with stages 1–3A accounting for 84.2% of all detected cases, while advanced stages (3B–5) represented 15.8%. Stage 3A was the most frequently identified stage (39.9%), followed by stage 2 (29.2%) and stage 1 (15.2%), indicating that most patients were detected before progression to advanced kidney disease.

**Figure 5:**
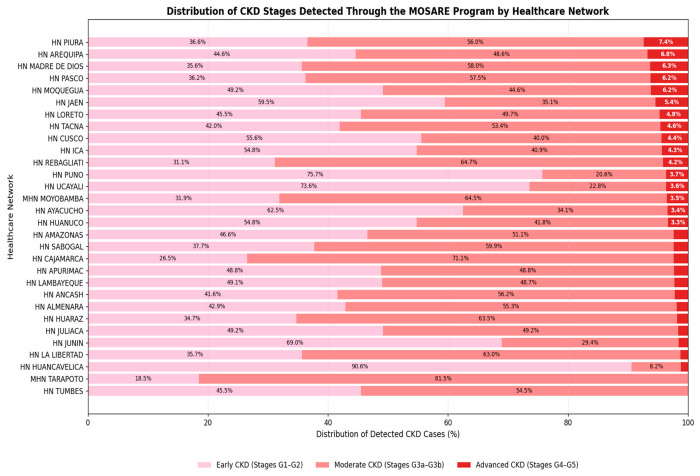
Distribution of patients screened using MOSARE within the Peruvian Social Health Insurance system, stratified by healthcare networks and departments, 2024.

Figure 5 shows the distribution of CKD stages across healthcare networks. Although stage distributions were generally consistent nationwide, relevant heterogeneity was observed. Networks such as Ucayali, Huancavelica, and Jaén showed a higher proportion of early-stage CKD, suggesting greater capacity for timely identification of kidney dysfunction. In contrast, Piura, Arequipa, Madre de Dios, Pasco, and Moquegua presented a relatively higher proportion of advanced CKD (stages G4–G5), indicating that a greater share of patients entered the care pathway at more advanced stages of the disease.

These findings demonstrate that MOSARE was effective not only in increasing CKD detection but also in identifying patients predominantly at earlier stages, thereby creating opportunities for risk-factor control, nephroprotective interventions, and delayed progression to kidney failure.

### 3.6. Sustainability

Sustainability analysis showed mixed results. Approximately 65% of trained facilities maintained active use of MOSARE (I-R1573) (Table 3), supported by ongoing training and technical assistance. However, challenges included interoperability issues and limited dedicated human resources. Increased screening trends in 2024 suggest continued system uptake, particularly in primary care, although differences across levels of care persist.

## 4. Discussion

In alignment with our primary objective to evaluate the nationwide implementation of the Renal Health Module (MOSARE) within EsSalud, the findings demonstrate a substantial operational scaling integrated into the national electronic health record system. When structured through the RE-AIM framework, the key results fulfill the study objectives as follows: (1) Reach: The module identified a macro-eligible universe of 4,613,598 individuals, successfully screening 93,368 patients (2.02% coverage) with a marked geographic heterogeneity (0.11% in Tumbes to 10.41% in Tacna); (2) Effectiveness: MOSARE yielded a 34.3% CKD positivity rate (*n* = 32,004), identifying an additional 1769 advanced cases compared to historical RENDES projections (a 71.16% increase); (3) Adoption: The tool achieved an active integration rate of 66.8% across primary and secondary healthcare facilities (*n* = 245/367); (4) Implementation: Fidelity to clinical staging was highly sensitive, capturing 84.2% of newly detected cases in early, preventable stages (1–3A); (5) Maintenance: High institutional sustainability was observed, with 65% of trained facilities maintaining active data integration despite persistent interoperability and workforce bottlenecks.

The findings demonstrate that MOSARE achieved broad implementation across the Social Health Insurance system, reaching 66.8% of level I and II healthcare facilities. By defining the institutional target universe as the denominator (*N* = 4,613,598), this strategy expanded the baseline window of digital screening beyond conventional risk cohorts. Consequently, while the absolute screening volume achieved a high sensitivity in identifying early-stage cases, the relative nationwide reach was calculated at 2.02%.

From a strict implementation science perspective, the nationwide reach of 2.02% achieved by MOSARE must be critically interpreted as low, highlighting a major discrepancy between digital capability and real-world clinical execution. While integrating universal automated triggers within a centralized electronic health record (EsSI) successfully identified a massive eligible universe, the actual transition to complete laboratory screening was severely limited. This low reach reflects systemic implementation bottlenecks typical of middle-income health systems, including substantial geographic heterogeneity in peripheral laboratory equipment, a lack of dedicated human resources to respond to automated alerts, and varying levels of administrative clinical leadership across networks (as evidenced by the stark contrast between Tacna’s 29.59% and Tumbes’ 0.35% coverage). Consequently, future scaling of MOSARE cannot rely solely on software integration; it demands targeted structural investments to mitigate workflow friction in low-performing regional networks.

From a RE-AIM perspective, this specific gap is highly representative of real-world digital health scaling in middle-income countries. It reflects that establishing wide digital automated inclusion triggers within an electronic record system is technically feasible, yet achieving high active screening coverage remains tightly constrained by health-workforce training variability, peripheral laboratory infrastructure heterogeneities, and clinical workflow friction across different geographic networks.

During 2024, the strategy screened 93,273 individuals with CKD risk factors, representing 5.59% of the identified at-risk population. Among those screened, approximately one in three patients (34.3%) was diagnosed with CKD, and more than four out of five detected cases were identified in early stages (1–3A). These findings support the value of MOSARE as a large-scale digital screening strategy capable of identifying CKD before progression to advanced disease.

The high detection rate observed (34.3%) must be interpreted with caution and should not be confused with general population prevalence. This elevated yield is primarily a function of targeted screening, as the evaluated cohort was heavily enriched with older adults (88.3% over 55 years) with established cardiometabolic comorbidities (35.9% hypertension, 20.6% diabetes). Furthermore, without a systematic 3-month confirmatory test, this percentage likely includes acute kidney injury (AKI) episodes or transient fluctuations in eGFR, a common phenomenon in registry-based secondary analyses in real-world settings.

These findings are particularly relevant, as CKD stage is a critical determinant in clinical decision-making, resource allocation, and timely access to renal replacement therapies [17,18]. MOSARE enables early differentiation between patients requiring structured nephrology follow-up and those suitable for periodic screening, thereby improving healthcare system efficiency. This approach aligns with international recommendations that prioritize early CKD detection as a cost-effective strategy to reduce disease progression and mortality [19,20].

From a RE-AIM perspective, MOSARE demonstrated moderate population reach, achieving screening coverage of 5.59% among individuals with diabetes and/or hypertension, the main at-risk group [7]. Nevertheless, important geographic disparities were observed. Departments such as Tacna, Callao, Ucayali, Huánuco, Loreto, and Lambayeque achieved substantially higher coverage than the national average, whereas Tumbes, Ica, Huancavelica, and La Libertad showed limited reach. These differences likely reflect variations in implementation capacity, availability of trained personnel, local leadership, and integration of digital tools within routine clinical workflows [21,22]. Consequently, future scaling efforts should prioritize low-performing regions to reduce inequities in CKD detection.

Regarding implementation outcomes, MOSARE demonstrated a substantial capacity to identify individuals with CKD across different stages of disease progression, with more than 84% of detected cases corresponding to stages 1–3A. The number of CKD cases identified through MOSARE exceeded those reported through conventional surveillance systems (RENDES) during the previous five years, including a higher number of advanced-stage cases. However, these differences should be interpreted cautiously, as variations in screening intensity, case ascertainment, reporting practices, and temporal factors may have contributed to the observed findings. The incorporation of the KFRE model provided additional clinical information by enabling risk stratification of patients, with risk distributions comparable to those reported in previous studies [16,23,24,25]. Furthermore, among patients who initiated renal replacement therapy, the median time between screening and treatment initiation was 58 days. Although this interval appears shorter than that reported in some regional settings [26], the present study was not designed to establish causal relationships between MOSARE implementation and treatment timelines.

Recent advances in artificial intelligence and digital nephrology are creating new opportunities for risk prediction, clinical decision support, and population-based screening of chronic kidney disease. Although MOSARE was not designed as an AI-based platform, its integration with electronic health records, automated risk stratification, and nationwide implementation infrastructure provides a foundation for the future incorporation of predictive analytics and AI-assisted tools to support CKD prevention and management [27].

Regarding adoption, 66.8% of facilities integrated MOSARE into routine practice, although substantial variability across networks indicates differences in implementation capacity. Barriers included interoperability issues, limited human resources, and insufficient knowledge of CKD guidelines, particularly in lower-resource settings [28].

Implementation indicators suggest that MOSARE was applied according to protocol in facilities with successful integration, although data quality issues highlight the need to strengthen monitoring systems. In terms of sustainability, 65% of trained facilities maintained active use, supported by ongoing training and technical assistance. However, persistent interoperability challenges and limited dedicated personnel threaten long-term sustainability.

Despite its large scale, this study has several limitations inherent to its observational design based on secondary electronic registry data. First, regarding imprecision, the data quality relies strictly on the manual and heterogeneous entry of laboratory values across different regional networks, which led to the systematic exclusion of 15,998 inconsistent or incomplete records (Figure 1). This missingness may introduce a slight underestimation of the active digital coverage. Second, potential selection and ascertainment biases are present; because the database captures individuals actively interacting with the health system, the clinical metrics likely overestimate the true population-level prevalence of CKD within the screened cohort.

Third, several confounding factors may influence our RE-AIM metrics and must be considered. Clinically, individual patient comorbidities (such as the severity and duration of uncontrolled hypertension or diabetes) and lifestyle factors were not uniformly captured within the module, potentially confounding the risk stratification outputs calculated by the KFRE model. Institutionally, the marked geographic variability in ‘Reach’ and ‘Adoption’ (Table 2 and Table 3) is likely influenced by unmeasured structural and contextual factors, including local administrative leadership, regional budgetary allocations for laboratory reagents, varying levels of internet connectivity, workforce availability, and organizational readiness across healthcare facilities. Furthermore, socioeconomic disparities, urban–rural differences, laboratory accessibility, and variations in healthcare infrastructure were not systematically collected and therefore could not be formally evaluated. These contextual factors may partially explain differences in implementation performance observed across regions and healthcare networks.

Fourth, a major methodological limitation is the lack of longitudinal patient follow-up to confirm true chronicity. Because staging relied on single-encounter laboratory entries, the rate of false positives or transient eGFR drops remains unquantified, and our metrics reflect operational implementation performance rather than true clinical efficacy or hard endpoints. Consequently, while MOSARE proves to be an effective public health tool, its operational success cannot be isolated from these underlying clinical and institutional confounding variables [29].

## 5. Conclusions

The initial nationwide implementation of MOSARE within EsSalud demonstrates that it is a promising and structurally viable digital tool for early CKD screening, enabling wide identification of at-risk patients, primarily at early stages. The RE-AIM evaluation highlighted operational strengths in baseline reach and diagnostic detection sensitivity, alongside clear systemic limitations in adoption and long-term sustainability that are tightly coupled with heterogeneous technological infrastructure, interoperability gaps, and human resource constraints. Furthermore, while the integration of the KFRE model offers an actionable pathway for clinical risk stratification, further longitudinal studies are required to confirm its direct impact on definitive clinical outcomes and hard endpoints. Overall, MOSARE shows preliminary potential to serve as a supportive model for chronic disease surveillance in the Peruvian health system, provided that future strategies systematically address the identified technological, training, and data quality bottlenecks.

## Figures and Tables

**Table 1:** Clinical characteristics of patients with chronic kidney disease (CKD) risk factors treated at IPRESS facilities within the Social Health Insurance system, 2024.

Characteristic	Category	*N*	%
Sex	Male	746,196	44.7%
Sex	Female	921,660	55.3%
Age group	Children (0–11)	562	0.0%
Age group	Adolescents (12–17)	1291	0.1%
Age group	Young adults (18–29)	3608	0.2%
Age group	Adults (30–55)	190,360	11.4%
Age group	Older adults (>55)	1,472,033	88.3%
Diabetes	No	1,323,603	79.4%
Diabetes	Yes	344,253	20.6%
Hypertension	No	1,068,668	64.1%
Hypertension	Yes	599,188	35.9%
Diabetes + Hypertension	No	1,498,024	89.8%
Diabetes + Hypertension	Yes	169,832	10.2%

**Table 2:** Distribution of patients with CKD risk factors who were screened and registered in MOSARE at IPRESS facilities of the Social Health Insurance system, by department, 2024.

Overall/Department	With CKD	%	Without CKD	%	Total Screened Patients	%	Scope of Population at Risk	%
	*n* = 31,990		*n* = 61,283					
Peru	31,990	34.30%	61,283	65.70%	93,273	100.00%	1,667,856	5.59%
Amazonas	378	55.34%	305	44.66%	683	0.73%	9399	7.27%
Ancash	1307	25.62%	3795	74.38%	5102	5.47%	57,567	8.86%
Apurímac	172	21.29%	636	78.71%	808	0.87%	13,726	5.89%
Arequipa	929	39.48%	1424	60.52%	2353	2.52%	122,235	1.92%
Ayacucho	443	34.75%	832	65.25%	1275	1.37%	17,652	7.22%
Cajamarca	248	40.39%	366	59.61%	614	0.66%	31,360	1.96%
Callao	3621	34.55%	6860	65.45%	10,481	11.24%	40,754	25.72%
Cusco	180	19.65%	736	80.35%	916	0.98%	50,424	1.82%
Huancavelica	85	75.89%	27	24.11%	112	0.12%	7910	1.42%
Huánuco	717	27.80%	1862	72.20%	2579	2.77%	23,219	11.11%
Ica	115	22.59%	394	77.41%	509	0.55%	68,884	0.74%
Junin	812	28.16%	2072	71.84%	2884	3.09%	52,155	5.53%
La Libertad	860	46.51%	989	53.49%	1849	1.98%	108,530	1.70%
Lambayeque	2755	30.48%	6284	69.52%	9039	9.69%	87,584	10.32%
Lima	11,439	37.72%	18,888	62.28%	30,327	32.51%	697,448	4.35%
Loreto	1301	34.35%	2487	65.65%	3788	4.06%	34,345	11.03%
Madre de Dios	174	46.03%	204	53.97%	378	0.41%	5068	7.46%
Moquegua	614	32.75%	1261	67.25%	1875	2.01%	18,877	9.93%
Pasco	80	20.30%	314	79.70%	394	0.42%	11,222	3.51%
Piura	705	27.99%	1814	72.01%	2519	2.70%	84,928	2.97%
Puno	1466	38.90%	2303	61.10%	3769	4.04%	36,523	10.32%
San Martín	450	43.95%	574	56.05%	1024	1.10%	30,017	3.41%
Tacna	2166	30.32%	4978	69.68%	7144	7.66%	24,142	29.59%
Tumbes	11	24.44%	34	75.56%	45	0.05%	12,881	0.35%
Ucayali	962	34.28%	1844	65.72%	2806	3.01%	21,006	13.36%

**Table 3:** Observations on adoption and maintenance derived from meeting minutes and official reports of the MOSARE implementation team within the Social Health Insurance system, 2024.

RE-AIM Domain	Excerpt from Meeting Minutes or Official Documents
Adoption: acceptance by IPRESS	“97% (381) of level I and II IPRESS report in MOSARE, highlighting growth in the Southern (60%), Northern (52%), and Central (33%) macro-regions” (I-000006).
Adoption: barriers identified for the use of MOSARE in healthcare facilities	“Lack of knowledge of current renal regulations among interdisciplinary teams in level I and II IPRESS was identified” (I-POI1T24). “Technical failures persist in the integration of the MOSARE registry into electronic health records, which affects system continuity” (I-R1573).
Adoption: facilitators for the acceptance of MOSARE as a digital screening tool for chronic kidney disease	“Virtual and in-person training sessions achieved an 85% acceptance rate of the module in trained IPRESS” (I-R1573). “Ongoing technical support provided to key healthcare networks, such as Rebagliati and Almenara, facilitated integration of the module into workflow processes” (I-POI1T24).
Maintenance: continued system use and integration of MOSARE into policy	“65% of reporting IPRESS maintained active use of the MOSARE module during the first half of 2024” (I-R1573). “Reporting IPRESS with advanced-stage cases improved their monitoring capacity through the use of the module, facilitating follow-up of critical cases” (I-R264).

IPRESS: Healthcare Service Provider Institutions.

## Data Availability

The raw data supporting the conclusions of this article will be made available by the authors on request because they contain individual-level health information derived from the EsSalud healthcare system. Access to these data is subject to institutional, legal, and ethical restrictions aimed at protecting patient confidentiality and privacy. The disclosure or redistribution of patient health information is regulated by EsSalud policies and by the Peruvian Personal Data Protection Law (Law No. 29733 and its regulations), which classifies health-related information as sensitive personal data requiring enhanced protection. Furthermore, Peruvian health regulations establish strict confidentiality obligations regarding medical information and prohibit the release of identifiable patient records without proper authorization.
